# Supplementary material for: Impact of Different Light Conditions on the Nitrogen, Protein, Colour, Total Phenolic Content and Amino Acid Profiles of Cultured Palmaria palmata
Source: Foods. 2023 Oct 27;12(21):3940. doi: 10.3390/foods12213940 (PMC10647453; doi:10.3390/foods12213940)
Supplement: Supplementary file 1 [file foods-12-03940-s001.zip › foods-2685277-supplementary.docx]

**Table S1** Pearson correlation analysis between colour difference (ΔΕ) and total nitrogen (TN), protein nitrogen (PN), non-protein nitrogen (NPN) and total phenolic content (TPC) of *Palmaria palmata* biomass cultured under different light treatment conditions. Whole fronds of *P. palmata* were grown at a density of 4 g/L with fertiliser (F/2) and with a 16:8 Light:Dark photoperiod at 100 µmol/m^2^/s photosynthetic active radiation (PAR) provided by different light-emitting diode sources: white (1 and 2), red, blue, green light using a Phillip’s Growise™ Control System for 6 and 12 days.

| **Correlation variables** | | **Pearson correlation coefficient**  **(statistical significance)** |
| --- | --- | --- |
| ΔΕ | TN | r = -0.230 (p = 0.522) |
| ΔΕ | PN | r = -0.125 (p = 0.731) |
| ΔΕ | NPN | r **= -**0.202 (p = 0.576) |
| PN | NPN | r = 0.195 (p = 0.589) |
| TN | TPC | r = -0.211 (p = 0.558) |
| PN | TPC | r **=** 0.246 (p = 0.494) |
| NPN | TPC | r = 0.209 (p = 0.563) |
